# Discovery of novel biomarkers associated with myocardial fibrosis in chimpanzees (*Pan troglodytes*)

**DOI:** 10.3389/fvets.2026.1794729

**Published:** 2026-05-19

**Authors:** Rachel Jarvis Martin, Kerstin Baiker, Sophie Moittié, Phillipa Dobbs, Kate White, Matyas Liptovszky, Melissa M. Grant

**Affiliations:** 1 School of Veterinary Medicine and Science, University of Nottingham, Sutton Bonington, United Kingdom; 2 School of Dentistry, Institute of Clinical Science, University of Birmingham and Birmingham Community Healthcare Foundation Trust, Birmingham, United Kingdom; 3 Veterinary Pathology Group, Bristol, United Kingdom; 4 School of Veterinary Medicine, St. George’s University, University Centre Grenada, West Indies, Grenada; 5 Veterinary Services, Twycross Zoo, Atherstone, United Kingdom

**Keywords:** biomarker, cardiovascular disease, chimpanzee, fibrosis, great ape, health, zoo

## Abstract

**Background:**

Idiopathic myocardial fibrosis (IMF) is a prevalent and life-threatening condition in captive chimpanzees (*Pan troglodytes*). Ante-mortem diagnosis remains challenging due to the limitations of current veterinary diagnostics. This study aimed to identify and validate circulating serum protein biomarkers for the detection of IMF.

**Methods:**

Serum samples collected from zoo-housed chimpanzees with post-mortem confirmed cardiac phenotypes were utilized. An initial discovery phase using a Proximity Extension Assay (PEA) screened 92 cardiovascular proteins in a subset of 10 chimpanzees. Three candidate biomarkers (ICAM-2, AXL, and PECAM-1) were subsequently selected for Enzyme-Linked Immunosorbent Assay (ELISA) validation in a broader cohort (*N* = 25). Final biomarker efficacy was assessed alongside Receiver Operating Characteristic (ROC) curve analysis.

**Results:**

In the discovery phase, ICAM-2, AXL, and PECAM-1 were significantly elevated in IMF-affected animals. During ELISA validation, circulating ICAM-2 remained significantly elevated in the IMF cohort (*p* = 0.010, Cohen’s *d* = 0.91). No significant association was detected with subject age or the time interval between sampling and death. AXL and PECAM-1 did not reach statistical significance in the validation cohort. ROC analysis for ICAM-2 established an optimal diagnostic cut-off of >1.535 ng/mL (AUC = 0.672), which demonstrated 100% specificity and a 100% positive predictive value.

**Conclusion:**

ICAM-2 is a highly specific, putative “rule-in” biomarker for moderate-to-severe IMF in chimpanzees. Implementing this biomarker into routine health assessments could enhance the early, non-invasive detection and clinical management of cardiovascular disease in this endangered species, though further validation is required before wider clinical use.

## 1 Introduction

Cardiovascular disease (CVD) is one of the leading causes of death in great apes (*Hominidae*) (1–3). However, despite the close genetic relatedness between humans and non-human great apes, their specific CVD phenotypes differ considerably (4). A leading cause of mortality in captive, endangered chimpanzees (*Pan troglodytes*) is a cardiomyopathy with an undetermined cause termed Idiopathic Myocardial Fibrosis (IMF) (3, 5–8). IMF is a chronic, degenerative condition in which the heart muscle is gradually replaced by excessive depositions of scar tissue, often resulting in sudden cardiac death due to terminal dysrhythmia (8, 9). Given that wild chimpanzee populations are diminishing, efforts to manage and improve their health and wellbeing in human care are increasingly necessary (10).

IMF has been identified as a major cause of mortality in the European and North American populations of chimpanzees in human care (1, 3, 4, 7, 8, 11). Multi-disciplinary teams of researchers and veterinarians such as the Ape Heart Project (Twycross Zoo and University of Nottingham, UK, linked with the present study), the Great Ape Heart Project (Zoo Atlanta, USA), and the International Primate Heart Project (Cardiff Metropolitan University, UK) have been collecting clinical and histopathological data on great ape CVD for over a decade with significant findings (12–15). Despite its prominence and previous investigations, the etiology of IMF remains unclear. Males appear to be at higher risk, and although fibrosis can be age-related, IMF also affects young individuals (3, 16). Viral infections, poor diet, diabetes mellitus, and vitamin E and selenium deficiencies have been reasonably ruled out (7, 8, 11, 17). However, genomic and environmental factors, as well as other metabolic deficiencies such as vitamin D, continue to be explored (12, 18).

While understanding the aetiopathogeneses of IMF is key to establishing appropriate treatment pathways, the ability to reliably detect the disease ante-mortem is essential for applying them effectively. Currently, cardiac ultrasound is the primary diagnostic tool used in zoological institutions. However, echocardiography is typically performed under general anesthesia, which inherently alters cardiac function and introduces cardiorespiratory variables depending on the protocol used (19, 20). Moreover, the cardiorespiratory effects depend on the exact anesthetic protocol used and the time since administration, which often varies between procedures and institutions (21, 22). Furthermore, routine cardiac ultrasound struggles to detect the microscopic tissue changes characteristic of early fibrosis. Consequently, the ‘gold standard’ method of diagnosing IMF remains a detailed post-mortem examination of the cardiac tissue (15).

To reduce IMF-associated mortality, safe and effective ante-mortem diagnostic tests are urgently needed. Conscious blood sampling is increasingly favored in zoo medicine as it is significantly less invasive and carries fewer health risks than general anesthesia, making serum-based diagnostic tools highly appropriate for this species.

Although well-established serum biomarkers such as cardiac troponin I (cTnI) and N-terminal pro-brain-type natriuretic peptide (NT-proBNP) are highly useful in human and veterinary medicine, their appropriateness varies considerably between species and disease phenotypes (23–28). Because cTnI and NT-proBNP are primarily designed to diagnose acute myocardial infarction and advanced heart failure, respectively, they may not accurately reflect the distinct fibrotic pathways of IMF in chimpanzees. Furthermore, previous pilot studies exploring alternative circulating biomarkers for the ante-mortem diagnosis of CVD in chimpanzees have yielded mixed prognostic utility, highlighting the need for a more targeted and species-specific approach (23, 28, 29).

To our knowledge, this is the first study to investigate circulating biomarkers for chimpanzee CVD using a rigorously paired biobank, matching ante-mortem serum directly with post-mortem histopathological diagnoses. This approach is widely considered the most accurate way to determine if a circulating biomarker reliably predicts cardiovascular morbidity (30). To discover novel candidates, this study utilized the Olink® Proximity Extension Assay (PEA), a modern, targeted proteomics method that screens for highly specific disease biomarkers (31–34). While initial high-content analysis (such as that used by Olink) is important for biomarker discovery, translating these findings into routine veterinary practice requires more accessible formats. Therefore, candidate biomarkers identified via PEA were subsequently evaluated using commercially available Enzyme-Linked Immunosorbent Assays (ELISAs), which represent a crucial step toward real-time, point-of-care diagnostics. Ultimately, the aim of this study was to identify and validate novel, highly specific serum biomarkers capable of diagnosing IMF in captive chimpanzees.

## 2 Methods

### 2.1 Study subjects

Twenty-five zoo-housed chimpanzees (*Pan troglodytes*) with a known cardiac phenotype were included as the subjects for this study. Opportunistically collected serum samples from these individuals were utilized for all subsequent biomarker analyses. Cardiac phenotype was determined according to the findings of standardized post-mortem cardiac examinations carried out by a board-certified veterinary pathologist in association with the Ape Heart Project, the methods for which have been outlined previously (15). Control animals were defined as those that died of non-cardiac cause, and where histological evaluation noted no to mild interstitial or replacement fibrosis. Affected animals were defined as those with moderate to severe replacement and/or interstitial fibrosis as described previously (8).

For biomarker discovery, an initial subset of 10 chimpanzee samples (Study A) was analyzed, followed by an additional 15 samples in a later validation phase (Study B, total *n* = 25, including all 10 samples from Study A). Study A included seven males and three females, with ages at death ranging from 18 to 62 years (median = 35). Six were classified as Affected (diseased) and four as Control (healthy). The time between serum sampling and death ranged from 0 to 35 months (median = 0). Study B included 11 males and 14 females, aged 9 to 62 years (median = 38). Of these, 17 were Affected and eight were Controls. The time between serum sampling and death ranged from 0 to 50 months (median = 0). A summary of information regarding all study subjects and their disease classifications can be found in Table 1. To maintain anonymity while allowing individuals to be tracked across both studies, each chimpanzee was assigned a unique alphanumeric identifier (Study ID).

**Table 1:** Study subjects: chimpanzees with a known cardiac phenotype, as confirmed histologically, categorized as control (healthy) vs. affected (diseased).

Study group		Study ID	Age at death (years)	Sex	Time between serum sample and death (months)	Disease phenotype
Study A (*n* = 10)	Study B (*n* = 25)	C2	37	Male	12	Affected
		C3	33	Male	1	Affected
		C4	33	Male	0	Affected
		C15	46	Female	0	Affected
		C18	18	Male	0	Affected
		C30	21	Male	14	Affected
		C9	32	Female	35	Control
		C31	62	Female	9	Control
		C20	21	Male	0	Control
		C26	28	Male	0	Control
		C8	39	Male	50	Affected
		C10	24	Female	7	Affected
		C17	45	Male	27	Affected
		C24	46	Female	0	Affected
		C29	44	Female	0	Affected
		C35	39	Female	19	Affected
		C41	43	Female	0	Affected
		C42	42	Female	0	Affected
		C44	32	Male	12	Affected
		C46	49	Female	26	Affected
		C47	47	Female	0	Affected
		C1	9	Male	0	Control
		C11	42	Female	0	Control
		C25	21	Female	0	Control
		C36	10	Female	0	Control

Study A (*n* = 10) was used for initial biomarker screening via Proximity Extension Assay (Olink® Target 96 Cardiovascular Panel III, v.6112). Study B (*n* = 25) included all individuals from Study A with an additional 15 chimpanzees and was used for subsequent ELISA testing.

Ethical approval for this study was obtained via the Committee for Animals and Research Ethics at the School of Veterinary Medicine and Science, University of Nottingham, UK (approval number SVMS 3064 200,106 and 2,253 18,322 Non ASPA VSA). All chimpanzee blood samples were collected opportunistically according to the Veterinary Surgeons’ Act (VSA) or international equivalent. Blood samples were centrifuged and separated before being stored frozen at −20 °C or below. All appropriate permits and licenses were obtained before samples were transported from the zoos of origin (*n* = 15 zoos) with freezer packs as coolant. Long-term storage of all samples was at −80 °C. On occasion, pooled human serum was used for ELISA quality control and optimization (approval number 19/SW/0198 Dental Research Tissue Bank at University of Birmingham’s School of Dentistry).

### 2.2 Proximity extension assay biomarker discovery (Study A)

Serum samples (250 μL per sample) from Study A were sent on dry ice to a commercial laboratory (Olink Proteomics, Uppsala, Sweden). 1 μL per sample was required for analysis using the high-performance Proximity Extension Assay (PEA) with a targeted panel of 92 protein biomarkers (Olink® Target 96 Cardiovascular Panel III, v.6112) to determine which proteins were significantly altered in chimpanzees affected by IMF (*n* = 6) versus healthy controls (*n* = 4). PEA readout values were in the form of Normalised Protein eXpression (NPX) on a log 2 scale, which corresponds to relative protein concentrations within the sample.

### 2.3 Enzyme-linked immunosorbent assay validation (Study B)

Enzyme-Linked Immunosorbent Assays (ELISAs) were used as subsequent protein measurements to validate the findings of the PEA discovery analysis on serum samples from animals in Study B. The following ELISA kits, containing the basic components necessary for the development of sandwich ELISAs, were assessed for use with chimpanzee serum: Human ICAM-2 Matched Antibody Pair Set (Sino Biological Inc., China, Cat# SEK10332, RRID: AB_3675613), Human Axl DuoSet (R&D Systems Inc., USA, Cat# DY154, RRID: AB_3675610) and Human CD31/PECAM-1 DuoSet (R&D Systems Inc., USA, Cat# DY806-05, RRID: AB_3675612). All assays were carried out at room temperature at University of Birmingham’s School of Dentistry, Institute of Clinical Sciences (5 Mill Pool Way, Birmingham, UK), using 96-well plates with a Nunc MaxiSorp surface treatment (Thermo Scientific Cat# 442404). An automatic plate washer was used for all wash steps, and a 2 N solution of sulphuric acid (H_2_SO_4_) was used to stop the reactions at the final stage, prior to determining the optical density (OD) using a microplate reader at 450 nm and 570 nm to allow for wavelength correction. The strength of the color changes of the samples corresponded to the concentration of the analyte, and each plate included a seven-point standard curve using two-fold serial dilutions of the high standard received in each kit. The substrate solution used in all cases was a 1:1 mixture containing two color reagents: Phosphate–Citrate Buffer with Urea Hydrogen Peroxide (Merck Life Science Cat# P4560) and 3,3′,5,5’-Tetramethylbenzidine Dihydrochloride (Merck Life Science Cat# T3405) dissolved in 10 mL deionised water. All solutions and buffers were freshly made up prior to the commencement of ELISAs.

The OD readout data from the assays were processed and transformed using Microsoft Excel. The final OD value for each sample was obtained by subtracting the 570 nm reading from the 450 nm reading. This correction ensures that only specific signals related to the analyte of interest are considered, minimizing any background absorbance signal. A seven-point standard curve using known concentrations from serial dilutions of the standards was plotted on a logarithmic scale. Using the line of best fit regression equation from the standard curve (y = mx + c), OD data of the test samples were converted into analyte concentrations with any dilution factors then accounted for.

The ICAM-2 kit was stored at −20 °C and contained the following reagents: rabbit anti-human capture antibody, rabbit anti-human detection antibody conjugated to horseradish-peroxidase, and a recombinant human standard. The standard was reconstituted with a detection antibody dilution buffer (0.5% bovine serum albumin [BSA] and 0.05% Tween20 in Tris-buffered saline [TBS]), and the other kit reagents were diluted to a working concentration using citrate-buffered saline or detection antibody dilution buffer, as per the manufacturer’s instructions. Plates were first coated with 100 μL capture antibody per well and incubated overnight at +4 °C. The following day, the plates were washed six times using 400 μL wash buffer (0.05% Tween20 in TBS) per well. Plates were blocked by adding 300 μL of blocking buffer (2% BSA and 0.05% Tween20 in TBS) per well and incubated for a minimum of one hour at room temperature prior to washing again. Standards diluted with sample dilution buffer (0.1% BSA and 0.05% Tween20 in TBS) and samples (100 μL per well) were added to the plate in duplicate and incubated for 2 hours at room temperature prior to washing. 100 μL of the detection antibody was added to each well and incubated for one hour at room temperature. After a final wash step, 200 μL of the substrate solution was added to the wells and incubated at room temperature away from light for 20 min, or until the standards on the plate showed a defined color change gradient. To stop the reactions, 50 μL of the stop solution was added prior to measuring the OD.

The AXL and PECAM-2 kits were from the same manufacturer (R&D Systems Inc., USA) and therefore the overarching protocols were the same. Both kits were stored at +4 °C and contained mouse anti-human capture antibody, biotinylated goat or sheep anti-human detection antibody, streptavidin conjugated to horseradish-peroxidase, and a recombinant human standard. All components were initially reconstituted, and later diluted to their working concentrations, with either neat phosphate-buffered saline (PBS) or PBS with added carrier protein (reagent diluent, 1% BSA in PBS), as per the manufacturer’s instructions. Plates were first coated with 100 μL capture antibody and incubated overnight at room temperature. The following day, the plates were washed six times using 400 μL wash buffer (0.05% Tween20 in PBS) per well. Plates were blocked by adding 300 μL of reagent diluent per well and incubated for a minimum of one hour at room temperature prior to washing again. Standards and samples (100 μL per well) diluted in the chosen reagent diluent were added to the plate in duplicate and incubated for 2 hours at room temperature prior to washing. 100 μL of the detection antibody was added to each well and incubated for 2 hours at room temperature. After washing, 100 μL of streptavidin conjugated to horseradish-peroxidase was added to each well and incubated for 20 min at room temperature away from light. After a final wash step, 100 μL of the substrate solution was added to the wells and incubated at room temperature away from light for 20 min, or until the standards on the plate showed a defined color change gradient. To stop the reactions, 50 μL of the stop solution was added before measuring the OD.

### 2.4 Quality control and optimization

Prior to using chimpanzee serum samples for final analyte measurements, assay performance was verified and optimized. Optimization of commercially available ELISA kits is often necessary to account for specific sample matrices, such as serum, which may introduce unique variables affecting assay performance. This practice is common to ensure accuracy and reliability in diverse research contexts (35, 36). Using pooled human serum as well as serum from one chimpanzee (study ID: C47) from whom there was a large volume of serum remaining, quality control tests including inter- and intra-assay variation, limit of detection (analytical sensitivity), range-finding, and spike-and-recovery were carried out for each ELISA kit (Thermo (35, 37)).

When sample replicates were *n* = 3 or above, inter- and intra-assay variation were calculated as the coefficient of variation (CV%), derived from the standard deviation divided by the mean, multiplied by 100 (35, 36). Analytical sensitivity (limit of detection) was calculated by adding two standard deviations to the mean OD obtained from 30 replicates of the zero standard (reagent diluent with no added sample). The range-finding exercise was conducted in order to assess at what dilution factor, if any, the analyte concentration measurement was stable and within the mid-range of the standard curve (36). By plotting analyte concentration (adjusted for dilution factor) against the dilution factor, the point at which dilution had little effect on measured concentrations (indicated by a plateau) was identified.

Then, a spike-and-recovery assay was performed using the sample dilution factor, where applicable, as determined by the range-finding exercise. For this, a known amount of analyte was added (spiked) into the test sample matrix and compared to an identical spike in the standard diluent. Where sample dilution was necessary, two different reagent diluents were tested for recovery: PBS with carrier protein (1% BSA) or without. This approach evaluates whether components in the sample matrix (e.g., serum) affect assay response differently than the standard diluent, aiming to maximize the signal-to-noise ratio and ensure consistent responses for the same analyte concentration in both matrices, allowing for reliable test sample measurement from the standard curve (Thermo (37)). If the average recovery falls within 80–120%, it indicates that the matrices have minimal impact on the assay’s accuracy (74). The recovery (%) was calculated using the following equation (35):


$$\frac{\left(Assayresultforspikedsample\right)-\left(Assayresultforneatsample\right)}{Amountspiked}*100$$

After these quality control and assay performance verifications, optimization of the sample dilution factor and reagent diluent used was carried out prior to final sample measurements (36). When final desired assay conditions were reached, all chimpanzees (Study B, *n* = 25) were tested for the three analytes ICAM-2, AXL and PECAM-1.

### 2.5 Data analysis

Data were analyzed using GraphPad Prism v10.3.0^1^ and jamovi 2.5.3 (The jamovi project, 2025).^2^ The significance threshold was set to *p* < 0.05 in all cases. All data were checked for normality using a Shapiro–Wilk test, which is appropriate for sample sizes <50 (77), and outlier analysis was performed prior to each statistical test. Where data were not normally distributed, non-parametric Mann–Whitney tests were used. Where data were normally distributed, parametric Welch’s *t* tests for independent samples were used to test for significant differences between chimpanzees affected by IMF and healthy controls. One-tailed tests were selected based on the hypothesis that analytes in the PEA cardiovascular panel would be raised in the diseased individuals, allowing for increased statistical power by focusing on the direction of expected change (75). To evaluate the magnitude of the biological difference between cohorts, effect sizes for significant parametric results were quantified using Cohen’s d.

The data from Study A were in the form of NPX (normalised protein) values and were already normalised on a log2 scale, meeting the assumptions of parametric tests. The Area Under the Curve (AUC) value for corresponding Receiver Operating Characteristic (ROC) curves was assessed for all 92 assay proteins. ROC curves are used to assess how well a diagnostic test can accurately distinguish between two conditions (e.g., affected vs. control), and the AUC is a numerical way to summarize the ROC curve’s performance (78, 79).

The data from Study B were in the form of protein concentration in ng/mL. Data were inspected in conjunction with each individual assay’s limit of detection (LOD). Left-censored values falling below the analytical LOD were conservatively substituted with LOD/2 prior to statistical analysis. Data were assessed for outliers using objective methodologies (e.g., Tukey’s interquartile range [IQR] method or GraphPad Prism’s ROUT method). Identified statistical outliers were investigated for technical or biological plausibility and excluded from final comparative analyses to prevent artificial skewing of the normative baseline. To rigorously address potential biological confounding factors, Spearman’s rank correlation coefficients (*ρ*) were calculated across the cohorts to evaluate the relationship between biomarker concentrations and both ‘age at sampling’ and the ‘time interval from sampling to death’. Furthermore, a targeted sub-analysis utilizing a Welch’s *t*-test was conducted exclusively on the restricted time-matched cohort (samples collected exactly at the time of death, time to death: 0 months) to assess biomarker dynamics absent of any temporal disconnect. ELISA data from Study B were evaluated for all 25 chimpanzees, as well as only the subset of 10 chimpanzees from Study A for a more direct comparison between the PEA and ELISA results.

### 2.6 Diagnostic value

Where statistical significance was reached with ELISA, the diagnostic value of a protein biomarker was assessed using Receiver Operating Characteristic (ROC) curve analysis. To establish the optimal diagnostic cut-off threshold, Youden’s Index (J) was calculated from the ROC curve data, where:


$$J_{1}=Sensitivity+Specificity-1$$

Sensitivity and specificity values were extracted at this optimal cut-off (80, 81). To further evaluate the clinical utility of the biomarker, the Positive Predictive Value (PPV) and Negative Predictive Value (NPV) were calculated based on the established optimal threshold. PPV was defined as the proportion of true positives among all positive test results:


$$PPV\;\left(\%\right)=\frac{TruePositives}{TruePositives+FalsePositives}*100$$

while NPV was defined as the proportion of true negatives among all negative test results:


$$NPV\;\left(\%\right)=\frac{TrueNegatives}{TrueNegatives+FalseNegatives}*100$$

## 3 Results

### 3.1 Proximity extension assay biomarker discovery (Study A)

**Figure 1:**
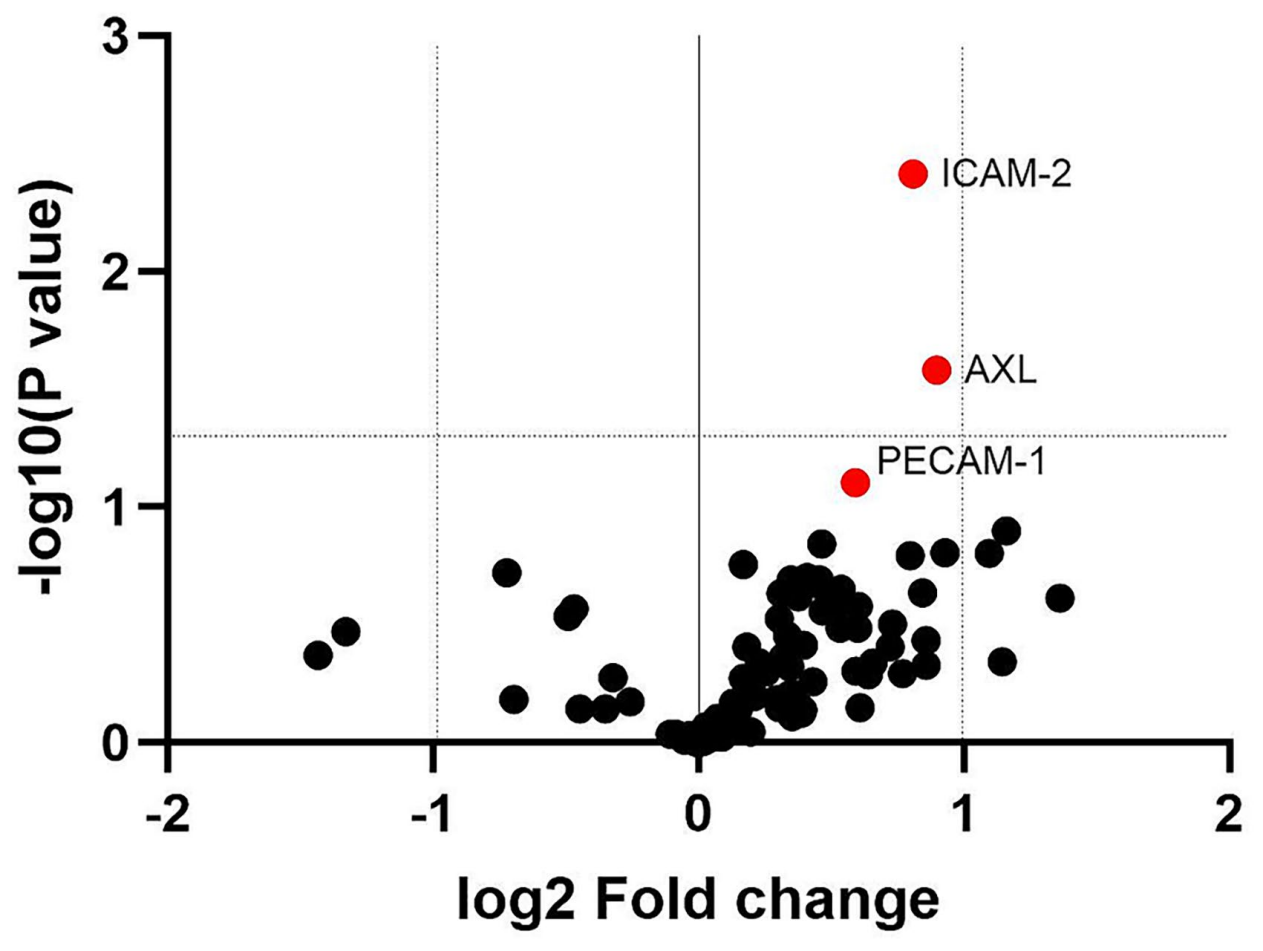
Volcano plot showing differences in protein expression (NPX) between chimpanzees affected by myocardial fibrosis (*n* = 6) and healthy controls (*n* = 4). The plot displays the −log10(*p*-value) (two-tailed Welch’s *t* tests) versus log2 fold change for 92 proteins (Olink® Target 96 Cardiovascular Panel III, v.6112). Proteins in red (ICAM-2, AXL, and PECAM-1) represent those selected for further exploration using ELISA, based on their significant differential expression as identified elsewhere. Dotted lines indicate thresholds for statistical significance (−log10[*p* value] > 1.3) and log2 fold change > 1 or < −1. Positive log2 fold change indicates upregulation in the diseased group, while negative values indicate downregulation.

**Figure 2:**
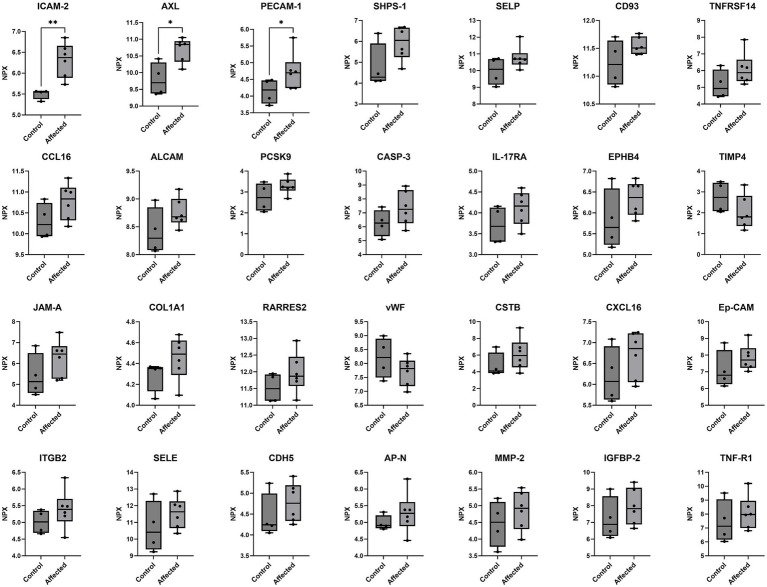
Normalized protein expression (NPX) of proteins in chimpanzees affected by myocardial fibrosis (*n* = 6) versus healthy controls (*n* = 4), as tested with proximity extension assay (Olink® Target 96 Cardiovascular Panel III, v.6112), which corresponds to relative protein concentrations within the sample on a log 2 scale. The 28 proteins with the highest area under curve (>0.7) out of 92 proteins tested in the assay are shown. Pairwise comparisons are displayed above each box-and-whisker plot where statistical significance is reached (one-tailed Welch’s *t* tests, **p < 0.01, *p < 0.05).

All of the 92 analytes tested in the serum screening were detectable in all samples, confirming cross-reactivity with the analytes in the assay panel. Figure 1 shows a volcano plot for the benefit of visualizing the significance and direction of changes in all 92 proteins. Proteins with an AUC value of >0.7 (*n* = 28) were selected for further investigation. Parametric one-tailed Welch’s *t* tests for independent samples were used to assess whether any of the 28 proteins were significantly altered in Affected chimpanzees compared with Controls. Three proteins, which are involved in fibrosis and inflammation, were significantly increased in Affected chimpanzees compared with Controls: Intercellular Adhesion Molecule 2 (ICAM-2, *p* = 0.002), Receptor Protein-Tyrosine Kinase (AXL, *p* = 0.013), and Platelet Endothelial Cell Adhesion Molecule 1 (PECAM-1, *p* = 0.039). Table 2 shows the AUC and Welch’s *t* test values for the 28 proteins of interest, in order of *p* value significance, and a visual representation of the differences in expression between the Affected and Control groups can be found Figure 2.

**Table 2:** Results of one-tailed Welch’s *t* test for independent samples and area under curve (AUC) analysis for proteins (as detected with Olink® Target 96 Cardiovascular Panel III, v.6112).

Protein	*p*-value	AUC value
ICAM-2	0.002 **	1.000
AXL	0.013 *	0.917
PECAM-1	0.039 *	0.833
SHPS-1	0.063	0.875
CCL16	0.072	0.792
TNFRSF14	0.078	0.750
CASP-3	0.078	0.750
SELP	0.081	0.708
COL1A1	0.088	0.792
TIMP4	0.095	0.792
IL-17RA	0.099	0.792
ALCAM	0.102	0.750
RARRES2	0.102	0.708
PCSK9	0.111	0.792
JAM-A	0.115	0.708
CD93	0.116	0.708
ITGB2	0.121	0.708
CSTB	0.122	0.708
EPHB4	0.133	0.750
CXCL16	0.138	0.750
Vwf	0.146	0.708
AP-N	0.149	0.750
Ep-CAM	0.157	0.792
CDH5	0.176	0.750
SELE	0.184	0.708
MMP-2	0.193	0.708
IGFBP-2	0.197	0.708
TNF-R1	0.257	0.708

Only proteins with an AUC value of >0.7 are included (*n* = 28). Asterisks denote a significant difference (**p < 0.01, *p < 0.05) in protein expression (NPX) between chimpanzees affected by IMF (*n* = 6) and healthy controls (*n* = 4).

### 3.2 Enzyme-linked immunosorbent assay validation (Study B)

#### 3.2.1 Quality control and optimization

The ICAM-2 Matched Antibody Pair Set ELISA kit performed with an inter-assay variation (CV%) of 17%, and intra-assay variation of 7%. The analytical sensitivity was 0.191 ng/mL, which fell within the range of the standard curve stated by the manufacturer (0.0469–3 ng/mL). The range-finding exercise revealed that no dilution of serum samples was needed. ICAM-2 showed a mean recovery (%) of 91% overall, with 100% recovery in the control spike (a known amount of analyte added to the standard diluent), and 86% recovery in serum (a known amount of analyte added to undiluted serum).

The AXL DuoSet ELISA kit performed with inter- and intra-assay variations (CV%) of 10 and 4%, respectively. The analytical sensitivity was 0.033 ng/mL, which was below the lowest point of the standard curve range as stated by the manufacturer (0.0625–4 ng/mL). A 1:120 dilution was found to be effective at ensuring the concentration of AXL in serum fell within the mid-range of the standard curve, while ensuring no adverse dilution effect. Overall, AXL showed a mean recovery (%) of 106%. When using PBS (without BSA) as the reagent diluent, there was a 110% AXL recovery in serum and 113% AXL recovery in the control spike. For this reason, as well as the fact that it is recommended elsewhere to use a sample diluent with no carrier protein for the dilution of complex sample matrices such as serum [Thermo (37)], PBS was chosen as the reagent diluent for the 1:120 sample preparations.

The PECAM-1 DuoSet ELISA kit performed with inter- and intra-assay variations (CV%) of 10 and 3%, respectively. The analytical sensitivity was 0.140 ng/mL, which was below the lowest point of the standard curve range as stated by the manufacturer (0.156–10 ng/mL). A 10-fold dilution (1:10) was found to be appropriate for PECAM-1, which showed a mean recovery of 106% overall. Again, PBS (without BSA) was chosen as the optimal sample diluent, as it gave a PECAM-1 recovery of 101% in 1:10 preparations of serum. Table 3 shows a summary of all assay performance results.

**Table 3:** Assay performances for ICAM-2, AXL and PECAM-1 as tested via commercially available human ELISA kits.

Assay parameter	ICAM-2	AXL	PECAM-1
Range (standard curve)	0.0469–3 ng/mL	0.0625–4 ng/mL	0.156–10 ng/mL
Inter-assay CV%	17%	10%	10%
Intra-assay CV%	7%	4%	3%
Analytical sensitivity	< 0.191 ng/mL	< 0.033 ng/mL	< 0.140 ng/mL
Dilution factor	Undiluted	1:120	1:10
% Recovery	91%	106%	106%
Reagent diluent	N/A	PBS	PBS

The standard curve range is as stated by the kit manufacturers. Analytical sensitivity (limit of detection) was calculated by adding 2 standard deviations to the mean optical density obtained from assaying 30 replicates of the zero standard. The % Recovery is the mean spike-and-recovery from a control spike (a known amount of analyte added to the standard diluent) and spiked serum (a known amount of analyte added to the sample matrix).

#### 3.2.2 Protein expression

**Figure 3:**
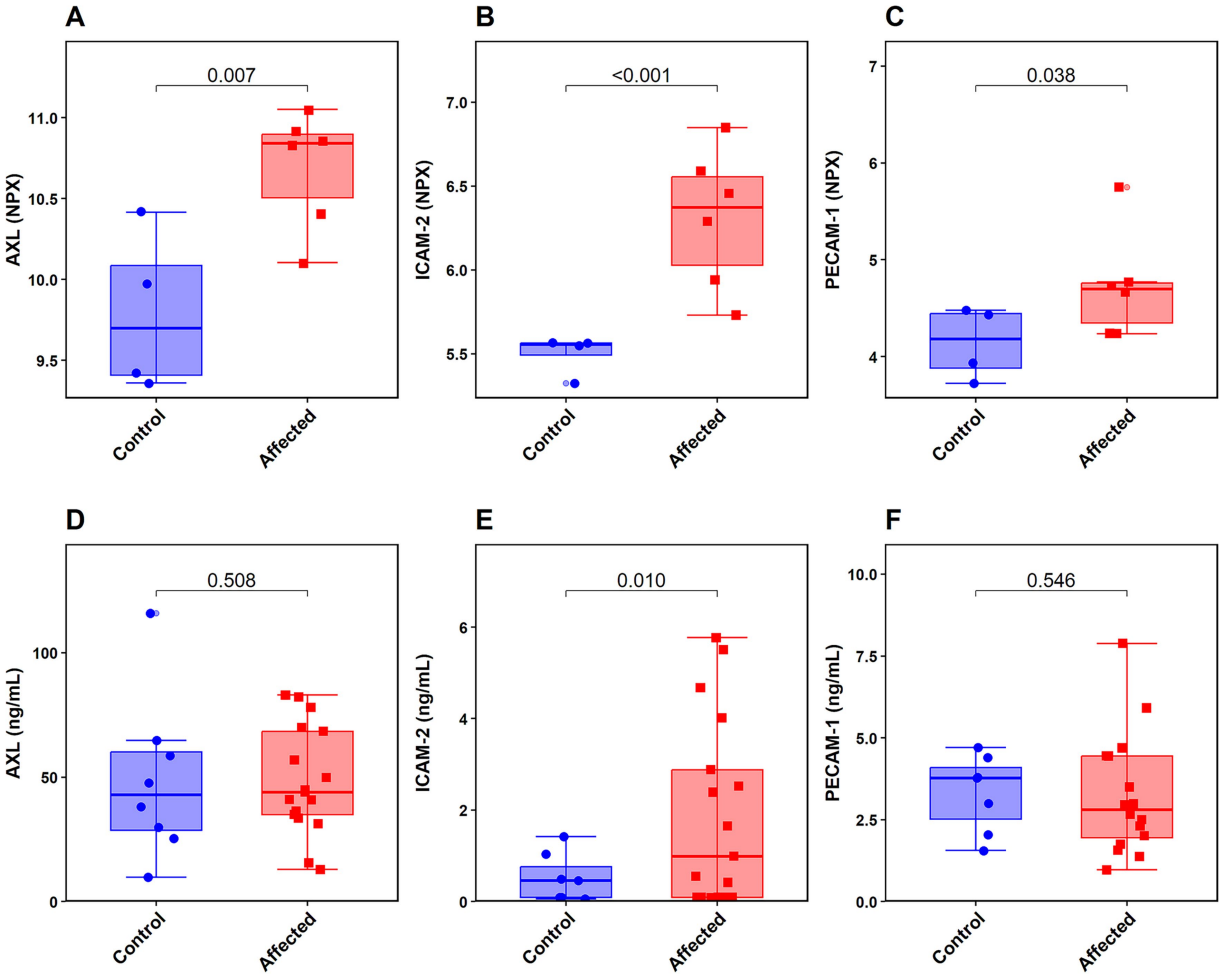
**(A–C)** Expression of AXL, ICAM-2 and PECAM-1 (*n* = 10) as tested by Olink Proximity Extension Assay (PEA) in the form of Normalised Protein eXpression (NPX), which corresponds to relative protein concentrations within the sample on a log 2 scale. Wilcoxon pairwise comparisons are displayed above each box-and-whisker plot. **(D–F)** Expression of AXL, ICAM-2 and PECAM-1 (in ng/mL) (due to assay-specific outlier exclusion) as tested by ELISA. One-tailed Welch’s *t*-test results are displayed above each box-and-whisker plot.

For ICAM-2, seven samples fell below the assay’s observed limit of detection (*n* = 1 Control, *n* = 6 Affected), these results were replaced with LOD/2 for statistical analysis. One sample was identified as an outlier (*n* = 1 Control) and was subsequently removed from analysis. In the final cohort (*N* = 24; 17 Affected, 7 Controls), the Affected group showed significantly elevated circulating concentrations of ICAM-2 (mean ± SD = 1.88 ± 2.04 ng/mL) compared to healthy controls (mean ± SD = 0.52 ± 0.53 ng/mL). A one-tailed Welch’s *t*-test confirmed this elevation was statistically significant [*t*(20.19) = 2.55, *p* = 0.010] (Figure 3), representing a large biological effect size (Cohen’s d = 0.91). To rigorously assess potential biological confounders, Spearman’s rank correlation revealed no significant relationship between ICAM-2 concentrations and age at sampling on the whole cohort (*N* = 24, rho = −0.063, *p* = 0.768), or the time interval from sampling to death within the Affected cohort (*N* = 17, rho = −0.101, *p* = 0.700). Additionally, a targeted sub-analysis of only those animals sampled at the time of death (time to death 0 months; *N* = 13) maintained a strong trend toward elevation in the diseased group (Welch’s *t*(8.36) = 1.68, *p* = 0.065). Crucially, the biological magnitude of this difference remained large (Cohen’s d = 0.853).

Conversely, AXL concentrations were not significantly different between Affected chimpanzees (*n* = 17, mean ± SD = 48.5 ± 21.6 ng/mL) and Controls (*n* = 8, mean ± SD = 48.8 ± 32.5 ng/mL) (Welch’s *t* test *p* = 0.508). No samples were excluded based on outlier identification for AXL.

With PECAM-1, two samples were identified as outliers (*n* = 1 Control, *n* = 1 Affected) and subsequently removed from analysis. PECAM-1 also did not differ significantly between Affected chimpanzees (*n* = 16, mean ± SD = 3.25 ± 1.84 ng/mL) and Controls (*n* = 7, mean ± SD = 3.32 ± 1.18 ng/mL) (Welch’s *t* test *p* = 0.546). When considering only the ELISA data from the initial subset of 10 chimpanzees (the same individuals as in Study A) in isolation, no statistically significant differences were found for any of the three proteins, though ICAM-2 approached significance: ICAM-2 (Mann–Whitney test *p* = 0.057), AXL (Welch’s *t* test *p* = 0.341) and PECAM-1 (Welch’s *t* test *p* = 0.387).

### 3.3 Diagnostic value

For ICAM-2, the only protein showing a significant difference between Affected and Control chimpanzees when measured with ELISA, diagnostic utility was assessed using ROC curve analysis. While the overall Area Under the Curve was moderate (AUC = 0.672, 95% CI: 0.451–0.893, *p* = 0.319), calculation of Youden’s Index established an optimal diagnostic cut-off of >1.535 ng/mL.

At this threshold, ICAM-2 demonstrated a specificity of 100% (correctly excluding all 7 healthy controls) and a positive predictive value (PPV) of 100%. Diagnostic sensitivity at this cut-off was 47.1% (correctly identifying 8 out of 17 diseased individuals), yielding a negative predictive value (NPV) of 43.75%. This provides strong initial evidence for ICAM-2 as a highly specific “rule-in” diagnostic biomarker for moderate-to-severe IMF, reflecting association with more advanced fibrosis.

## 4 Discussion

### 4.1 Proximity extension assay biomarker discovery (Study A)

This study has made use of histopathology paired samples and data from the international sample network driven and maintained by the Ape Heart Project. The PEA system uses very low sample volume meaning that valuable samples can be preserved for investigation in a wider range of experiments. In the initial PEA discovery, three protein biomarkers were identified as being significantly increased in serum in diseased chimpanzees with histologically confirmed myocardial fibrosis compared with their healthy counterparts. Though the PEA analysis used a panel of 92 proteins that are generally involved in human CVD, the three resultant candidates AXL, ICAM-2 and PECAM-1 have been indicated more specifically in inflammatory and fibrotic diseases in the wider literature.

AXL is a transmembrane tyrosine kinase receptor expressed ubiquitously in mammalian tissues with importance in normal cell functions such as the regulation of cell adhesion, inflammatory responses, and coagulation (38, 39). Overexpression of AXL has been indicated in various morbidities, including coagulopathy, autoimmune disease, and cancers (39, 40). Notably, AXL is also directly involved in fibrotic diseases of the kidney, intestines, liver and lungs (41–44), and can play a maladaptive role in cardiac remodeling and inflammation during CVD (45–48). Moreover, the use of AXL together with BNP has previously shown a better predictive value for heart failure events than using either marker independently (49), reinforcing the idea that the use of biomarker signatures is beneficial.

ICAM-2 is present on the surface of platelets and is an essential ligand in antigen-specific immune responses including the migration of immune cells during inflammation and thrombosis (50–52). ICAM-2 is upregulated during inflammatory lung diseases such as cystic fibrosis and idiopathic pulmonary fibrosis (53, 54) and has been implicated in vascular diseases and endothelial dysfunction during chronic heart failure (55, 56).

PECAM-1 is an endothelial adhesion molecule within the immunoglobulin superfamily that, similarly to ICAM-2, regulates thrombosis, cardiomyocyte contractility, transendothelial immune cell migration and endothelial cell function (57, 58). PECAM-1 expression is linked with myocardial damage following ischaemic events, and its genetic polymorphisms are linked directly with diseases of the coronary vasculature and acute cardiac events in humans (59–61). Moreover, PECAM-1 has been directly indicated in the disease progression of liver fibrosis (62).

Interestingly, the widely used cardiac biomarker NT-proBNP was not significantly altered in this group of diseased chimpanzees. Cardiac troponin, however, was not included in the PEA assay panel and was therefore not tested.

### 4.2 Enzyme-linked immunosorbent assay validation (Study B) and clinical implications

The use of ELISA set out to validate the findings of the PEA discovery by using a secondary analysis via commercially available assay kits that are for use with human biological samples. It was possible to expand the number of samples analyzed due to the time elapsed between the PEA discovery analysis and the validation by ELISA as more samples had been donated to the biobank at this point. Of the three markers, ICAM-2 was the only analyte to reach significance between the groups when tested with ELISA (*p* = 0.010), representing a large biological effect size (Cohen’s d = 0.91) and thus confirming the findings of the PEA discovery. Further sub-analyses confirmed this elevation was independent of subject age or the time interval between sampling and death. PECAM-1 and AXL did not differ significantly between groups in the broader validation cohort.

Evaluation of ICAM-2 via ROC curve analysis established an optimal diagnostic cut-off of >1.535 ng/mL, yielding a specificity of 100% and a sensitivity of 47.1%. From a clinical perspective, this high specificity is exceptionally valuable. It indicates that ICAM-2 is highly effective at correctly identifying individuals affected by moderate to severe IMF, functionally eliminating the likelihood of false-positive results in this cohort. In zoological medicine, comprehensive cardiovascular phenotyping typically requires general anesthesia, which carries inherent morbidity and mortality risks, particularly in great apes with suspected cardiac compromise (76). A 100% specific blood test allows veterinary teams to confidently “rule-in” IMF using opportunistically collected conscious or minimally invasive blood draws. Animals exceeding the 1.535 ng/mL threshold can be prioritized for comprehensive echocardiographic evaluation and therapeutic intervention, mitigating the risk of unnecessarily anesthetising healthy animals. Conversely, the negative predictive value (NPV) of 43.8% indicates that ICAM-2 cannot be used as a standalone test to definitively “rule-out” IMF, but it serves as a highly effective, targeted “rule-in” screening tool.

### 4.3 Disagreement between PEA and ELISA

The lack of absolute agreement between the findings of the PEA panel and the ELISAs in this case is not particularly unusual, as previous studies have found similar discordance (63, 64). This could be for a number of reasons, such as potential post-translational modifications being detected in one assay and not the other, the PEA having a higher degree of specificity than the ELISA kits, or differences in assay conditions such as sample volume (1 μL for PEA vs. 100 μL for ELISA) and dilution factors used (neat sample for PEA vs. diluted for ELISA) (65). It is also possible that pre-analytical factors may have affected the results. The ELISA testing took place several years after the PEA discovery, thus sample storage and transport conditions likely affected the quality of the samples. Separate aliquots of the serum samples were used for the PEA and ELISA testing, which may have affected protein concentrations in each assay if the samples were not fully homogenized prior to aliquot separation (65). While the absolute quantification of the analytes may vary between platforms in this case, it has been suggested that if both assay platforms perform well, the overall sample ranking should be similar (66), as has been displayed in the present study. There are several examples in the literature of strong positive correlations between PEA and ELISA, though often this does not apply to all proteins tested (67, 68).

### 4.4 Limitations

The performance of the ICAM-2 ELISA kit with chimpanzee serum showed an inter-assay variation of 17%, which is marginally higher than the commonly accepted threshold of 15%, and required several samples falling below the limit of detection to be substituted with an LOD/2 value. However, the intra-assay variation was acceptable at 7%, and the 91% analyte recovery was well within the acceptable boundaries of 80–120%.

The small sample size in this study can be considered a limitation. While PEA technology is a technically robust method, it has been suggested that moderate effect size proteins would require 400 samples per group (test vs. control) in order to obtain confidence in any statistical difference for an assay panel containing 100 test proteins (69). Despite this, it is important to consider the inherent challenges in obtaining samples from endangered wildlife species, including those housed in zoos and sanctuaries. In our case, all samples need to be collected opportunistically during routine and emergency veterinary procedures and the international network we have used to obtain paired samples (whole heart for post-mortem phenotyping in addition to an ante-mortem serum sample) has required extensive efforts. Additionally, there is a lack of absolute control over how the participating zoos store and transport their samples to the project, thus the quality of pre-analytical conditions is not always assured. It is hoped that the acquisition of paired samples will continue over time, and that future studies will have access to larger sample sizes for continued investigations into great ape cardiovascular health.

Furthermore, the opportunistic nature of sampling in zoological institutions resulted in variable time intervals between serum collection and death. While the implementation of a strict 50-month inclusion cap and a targeted sub-analysis of time-matched (0-month time gap) animals successfully confirmed that the diagnostic signal of ICAM-2 persists independent of temporal disconnects, it is hoped that the acquisition of paired samples will continue over time, and that future studies will have access to larger sample sizes or longitudinal datasets for continued investigations into great ape cardiovascular health.

Another limitation of this study is that the specific Olink PEA panel and ELISA kits used were designed and validated for human samples only. Though chimpanzees have long been used as animal models in biomedical research due to their strong genetic similarity to humans, the two species can vary considerably in their natural genetic and proteomic expressions in serum and tissue (70–73).

Furthermore, the pathological classifications of chimpanzees in our study presents its own challenges. Individuals affected by IMF may have co-morbidities such as concurrent infections that may lead to systemic inflammation, potentially confounding the specificity of circulating biomarkers of IMF. No macroscopic nor microscopic evidence for organ infections was found on detailed post-mortem examinations, but subclinical infections cannot be fully excluded. Chronic renal disease with the consequence of systemic hypertension and chronic cardiac remodeling has also been regularly found in captive great apes and the precise degree of overlap of IMF with changes caused by other chronic cardiovascular diseases remains a diagnostic challenge (22). There is a possibility that identified serum changes are confounded by non-specific myocardial fibrosis rather than the captive ape-specific IMF, highlighting the need for continued validation in larger cohorts.

## 5 Conclusion

This study identified and validated circulating ICAM-2 as a novel serum biomarker for moderate to severe idiopathic myocardial fibrosis (IMF) in captive chimpanzees. While the Proximity Extension Assay provided valuable initial insights into the inflammatory and fibrotic disease pathways of IMF, the subsequent validation of ICAM-2 via ELISA provides the most immediate clinical value. Demonstrating 100% diagnostic specificity ICAM-2 serves as a potential minimally invasive “rule-in” test capable of identifying high-risk individuals. Pending further validation, implementing this biomarker into routine health assessments in great apes in human care could allow veterinary teams to confidently prioritize affected animals for advanced cardiological evaluation and targeted therapeutics, while sparing healthy individuals from the inherent risks of general anesthesia. Ultimately, the clinical integration of such targeted diagnostics may represent a critical step toward the early detection and prompt treatment of cardiovascular disease, which has the potential to significantly lower the mortality rate of CVD-related deaths among zoo-housed chimpanzees.

## Data Availability

The raw data supporting the conclusions of this article will be made available by the authors, without undue reservation.

## Footnotes

1
https://www.graphpad.com/scientific-software/prism/

2
https://www.jamovi.org
